# Supplementary material for: Pathogenic Huntingtin aggregates alter actin organization and cellular stiffness resulting in stalled clathrin-mediated endocytosis
Source: eLife. 2024 Oct 9;13:e98363. doi: 10.7554/eLife.98363 (PMC11643626; doi:10.7554/eLife.98363)
Supplement: Supplementary file 1. [file elife-98363-supp1.docx]

**Supplementary file 1.**

Table showing the pixel and frame rate information for each cell type.

| **Cell Type** | **Pixel width in µm** | **Frame interval in seconds** |
| --- | --- | --- |
| WT | 0.0081 | 5.00 |
| HTT Q138 | 0.0195 | 5.00 |
| LatA treated | 0.0122 | 7.96 |
| DMSO treated | 0.0122 | 7.96 |
| Hip1 overexpression | 0.0081 | 8.82 |
| Profilin RNAi | 0.0033 | 5.00 |
| Arp2/3 overexpression | 0.0122 | 9.11 |
| Arp2/3 RNAi | 0.0037 | 5.0 |
| Hip RNAi | 0.0081 | 9.11 |
| Mrj overexpression | 0.0122 | 8.82 |
